# Adolescents’ Experiences, Emotions, and Coping Strategies Associated With Exposure to Media-Based Vicarious Racism

**DOI:** 10.1001/jamanetworkopen.2021.13522

**Published:** 2021-06-15

**Authors:** Nia Heard-Garris, Patricia O. Ekwueme, Shawnese Gilpin, Kaitlyn Ann Sacotte, Leishla Perez-Cardona, Megan Wong, Alyssa Cohen

**Affiliations:** 1 Division of Advanced General Pediatrics and Primary Care, Ann & Robert H. Lurie Children’s Hospital of Chicago, Chicago, Illinois; 2 Mary Ann & J. Milburn Smith Child Health Outreach, Research, and Evaluation Center, Stanley Manne Children’s Research Institute, Ann & Robert H. Lurie Children’s Hospital of Chicago, Chicago, Illinois; 3 Department of Pediatrics, Northwestern University Feinberg School of Medicine, Chicago, Illinois; 4 Department of Pediatrics, John H. Stroger Jr Hospital of Cook County, Chicago, Illinois

## Abstract

**Importance:**

Adolescents frequently encounter racism vicariously through online news and social media and may experience negative emotional responses due to these exposures. To mitigate potential adverse health impacts, including negative emotional health, it is important to understand how adolescents cope with these exposures.

**Objectives:**

To examine adolescents’ responses to online and media-based vicarious racism exposure and to explore coping strategies, particularly positive coping strategies, that may be used to combat negative emotions.

**Design, Setting, and Participants:**

This qualitative study rooted in phenomenological research methods conducted 4 semistructured focus groups, with 3 to 6 English-speaking adolescents (aged 13-19 years) in each group, between November 2018 and April 2019. Focus groups were facilitated by 2 research team members. The study was conducted at community sites and youth organizations in the greater Chicago, Illinois, area. Interview transcripts were analyzed thematically.

**Exposures:**

Lived experiences of media-based vicarious racism.

**Main Outcomes and Measures:**

Focus group participants shared their experiences with media-based vicarious racism online, including their responses to exposure and the coping strategies used.

**Results:**

Four focus group sessions were conducted with a total of 18 adolescents. Participants had a mean (SD) age of 16.4 (1.6) years. Overall, 7 participants (39%) self-identified as Black/African American, 8 (44%) as Hispanic/Latinx, and 3 (17%) as White individuals; 7 (39%) were in grades 7 to 9, 8 (44%) in grades 10 to 12 grade, and 3 (17%) at the college or university level. Central themes emerged related to adolescents’ experiences, including their emotional and coping responses to media-based vicarious racism. Many participants reported helplessness as a major negative emotion associated with these exposures. Activism was endorsed as a key positive coping strategy that participants used, including online and in-person modalities.

**Conclusions and Relevance:**

The findings from this qualitative study suggest adolescents may experience helplessness as a primary negative emotion after exposure to media-based vicarious racism and activism may serve as a coping mechanism. Activism may represent an important and constructive means by which adolescents cope with and combat structural racism, mitigate negative emotions, and potentially prevent adverse health effects.

## Key Points

**Question:**

How do adolescents respond to media-based vicarious racism, and do these responses affect adolescent emotional health and well-being?

**Findings:**

This qualitative focus group–based study with 18 participants found that adolescents experienced helplessness after exposure to media-based vicarious racism and that activism was used as a positive coping strategy.

**Meaning:**

The findings suggest that activism may serve as a powerful coping mechanism, potentially reducing negative emotions for adolescents exposed to media-based vicarious racism; thus, activism may have implications for improving mental health outcomes and advancing societal changes.

## Teaser

This qualitative study examines adolescents’ responses to online and media-based vicarious racism exposure and explores coping strategies, particularly positive coping strategies, that may be used to combat negative emotions.

## Introduction

Racism is entrenched in US institutions, with well-documented impacts on physical and mental health and well-being, even if experienced secondhand.^1,2,3,4^ Institutions such as health care, government, carceral systems as well as news and media are inextricable from the pervasive nature of racism. News and online platforms, such as social media, can be an important conduit for vicarious racism at the population level and may also affect health. Vicarious racism is the secondhand exposure to racism directed at another individual and occurs irrespective of the race of the unintended target; however, that unintended target or bystander must identify the event as racism.^4^ Recently, the disproportionate murders of members of racial/ethnic minority groups by police has been a type of structural vicarious racism within the public discourse and has been associated with negative repercussions for health.^5^

Adolescents consume news of racialized violence and other racially charged events that are disseminated through traditional and online media as well as social media outlets.^6^ Scholarship on media-based vicarious racism exposures is emerging, and qualitative studies have begun to describe negative emotions after media-based vicarious racism exposure in adolescents.^4,7,8^ Given the ubiquity of these stories and ease of internet access, adolescents may have more exposure to structural racism experienced vicariously than previous contemporary periods. Thus, youth may have an increased need for positive coping mechanisms to mitigate adverse health outcomes from this exposure.

Although structural racism may require intervention at the societal level, adolescents may not have traditional access to positions of authority or policy making. However, in today’s technology-driven and highly interconnected world, adolescents may seek out unique strategies to prevent and cope with racism. Given the nascent literature, studies have not yet explored how adolescents navigate media-based vicarious racism or identified the coping strategies that adolescents use following vicarious racism exposure.

Using an adolescent-centered, qualitative approach, we sought to (1) examine how adolescents respond to media-based vicarious racism and (2) to explore coping strategies adolescents use to mitigate negative emotions and structural racism, vicariously experienced in the media. We hypothesized that adolescents use a variety of coping skills after exposure to media-based vicarious racism. We also anticipated that adolescents would openly discuss the impact of vicarious racism on their health. Finally, structural racism and the resultant adverse health outcomes and disparities will require societal, institutional, and individual transformation. However, by focusing on individual-level experiences and strategies, adolescents may be able to actively lessen the negative consequences of racism on their own health and well-being.

## Methods

### Study Design

This study was part of a larger qualitative study that examined 3 major thematic categories: youth experiences with media, experiences with racism online, and responses to racism.^8^ The current study used a phenomenological approach and reflexive thematic analysis to produce descriptive data focused on adolescent responses to racism as a critical part of elucidating how adolescents are affected by racism.^9^ Phenomenological research aims to describe and find commonalities in the lived experiences of a concept or of a phenomenon, in this case, racism in news and online media as experienced by adolescents.^9^ Focus group interviews were used to offer adolescents an opportunity to verbalize their experiences among peers, creating open-ended discussions to allow deeper understanding of their perspectives. The focus group design has been used in prior research surrounding sensitive experiences, like racism, to lessen participants’ sense of discomfort or personal stigma.^8,10^ This study was approved by the Ann and Robert H. Lurie Children’s Hospital of Chicago institutional review board. This study followed the Consolidated Criteria for Reporting Qualitative Research (COREQ) reporting guideline.

### Study Participants

Adolescents from all racial and ethnic backgrounds, aged 13 to 19 years at the time of enrollment, and able to read and communicate proficiently in English were eligible to participate in this study. A community-based recruitment strategy was used with flyers posted in community sites, such as public libraries, public meeting spaces, and pediatric outpatient clinics associated with study members’ institutions. Additionally, the research team partnered with community youth organizations for recruitment. Focus groups were held in accessible private meeting areas in the greater Chicago, Illinois, area. Participants and their parents completed the assent and consent forms, respectively. Participants were advised that by signing the assent/consent, they were agreeing to have responses used in the study.

### Data Collection

A standardized semistructured interview guide was developed, comprising open-ended questions about participants’ interactions with social media, access to news media, experiences of racism through news and online media, and responses to such experiences. Questions were formulated based on extant literature and ongoing studies on these topics and were piloted for sequence and timing. The interview guide also included optional prompts with examples of racist events (eg, incidents of race-related police violence or deportation) and of health behaviors (eg, eating healthy foods and taking prescribed medications), if participants required clarification.

Four focus groups were held during the study period of November 2018 to April 2019, with 3 to 6 participants in each group (18 participants total). Each focus group was facilitated by 2 research team members experienced in focus group interviewing. These facilitators self-identified as Black, Pakistani, or White. Three focus groups included a mix of Black and Latinx/Hispanic adolescents, and these groups were led by a diverse group of facilitators. We used a diverse study team to encourage participants to share their experiences more freely. One focus group had primarily White adolescents, and this group was led by White facilitators. Before each focus group session, participants self-reported their age and educational level, and wrote in their self-identified race and ethnicity, summarized in Table 1. Participants used code names to ensure privacy. Research team facilitators posed open-ended questions to the whole group, provided clarifications as necessary, and encouraged each participant to respond to each question but also gave the option to decline any question or leave the group without any penalty. Focus groups (mean [SD] duration, 51.25 [28.72] minutes) were recorded digitally and transcribed using Descript transcription software (2019).

**Table 1.** Adolescent and Young Adult Participant Demographic Characteristics

Characteristic	No. (%) (N = 18)
Age, y	
Mean (SD)	16.4 (1.64)
13-15	7 (39)
16-17	4 (22)
18-19	7 (39)
Race/ethnicity	
Black/African American, non-Hispanic	7 (39)
Hispanic/Latinx	8 (44)
White, non-Hispanic	3 (17)
Education level	
Grade	
7-9	7 (39)
10-12	8 (44)
College or university	3 (17)

### Data Analysis

Focus group transcripts were compared to audio recordings for accuracy and underwent reflexive thematic analysis^11^ using the qualitative coding software Dedoose version 8.0.35. Three of us (A.C., P.O.E., and N.H.G.) and an additional researcher independently reviewed transcripts from each focus group, highlighting significant statements and identifying emergent codes. Through iterative review and triangulation among team members, codes were clustered or organized into themes and subthemes. Data analysis progressed in tandem with focus group interviews to assess for data saturation, and no new major themes had emerged at the time of the final session. Once finalized, themes were compared against the original transcripts, and select subthemes were further analyzed to understand the circumstances in which they occurred—ie, participants’ experiences of racism online. The study authors also considered their own positionality with respect to this topic, and the first author’s perspective is detailed in the eAppendix in the Supplement.

## Results

### Demographic Characteristics

Four focus group sessions were conducted with a total of 18 participants. Participants had a mean (SD) age of 16.4 [1.6]; 7 (39%) self-identified as Black/African American, 8 (44%) as Hispanic/Latinx, and 3 (17%) as White adolescents; 7 (39%) were in grades 7 to 9, 8 (44%) in grades 10 to 12, and 3 (17%) at the college or university level. Self-reported demographic information was collected from all participants prior to the start of the focus group in an open-ended manner (Table 1). Age, race/ethnicity, and gender identity were collected; however, gender identity was omitted from Table 1 to maintain anonymity for gender minority youth.

### Findings

This study focuses on adolescent responses to racism, with specific attention to emotional and coping responses. Participants described coping mechanisms, such as disconnecting from media, focusing on hobbies or being creative, talking with peers, and positive thinking. However, helplessness and activism were 2 central themes that emerged from the data analysis.

### Emotional Response: Helplessness Theme

Participants reported adverse emotional responses to racist events, both in-person and online. Helplessness, as described by participants, emerged as an important negative emotion. Helplessness was a salient theme and centered on statements such as “there’s nothing I can do” and included 3 subthemes (Table 2).

**Table 2.** Helplessness Theme and Examples of the Subthemes from Adolescent and Young Adult Participants

Theme	Subtheme	Examples of statements introduced by participants
Helplessness	Racism as a part of the world	[After witnessing racially-charged news] “Sometimes I don’t say anything. I’m just like okay. Another day in the life.”
		“I think it’s become more of a norm seeing this on the news and stuff. So, when I see it, it’s just like, oh this is happening again.”
		“I feel like that was the spot for me to be like, ‘Oh wow. This is the country we live in,’ so yeah, I feel like I’ve gone numb to when someone will be like, ‘Oh, rac[ism],’ I’ll just be like, ‘Okay, yeah, sure.’”
		“I’m trying to think of a good example, but the best way I can think of is the camps that the children are put into … I don’t understand our history, but I do think that as a people we are capable to be like, ‘Hey, this legal system isn’t working, let’s just trash it and do something else,’ and we don’t. We just keep listening to the same White men from 200 years ago, and that was 200 years ago, and now we have f**** watches … Sorry. We have watches that talk to us, so clearly the [old] rules are changing, not applicable. They are not.”
	Dependent on target of racism	“The only way that it could actually change my mood is if it’s to me. As opposed to if it happened in front of me and it happens to me, then I could subjectify the issue and actually get angry, but if it’s not towards me. I mean, I can talk about it and try to change the situation, but 10 times out of 10 … there’s nothing I can do.”
	Futility in responding	“Usually, unless it’s truly personal or it’s somebody that I know, I won’t get involved just because it’s a waste of time. Every time I think we’re making progress, I see another 13-year old on YouTube who commented something stupid. Those are the people that you just cannot change their mind because their minds don’t want to be changed.”
		“Again, I know that people are going to think whatever they’re going to think and they’re not going to change their minds until they’re ready or until they decide like, ‘Okay, I’m the one that is deciding it is wrong to be racist, not you telling me it’s wrong.’ People are going to do what they’re going to do, they’re going to say what they’re going to say.”

#### Subtheme 1: Racism as a Part of the World

Adolescents perceived that adults underestimated their awareness of the world around them and stated that they are not only acutely aware but witness the pervasive effects of discrimination and feel overwhelmed. Helplessness was typically the first response participants expressed regarding their emotional state after viewing racism on the news. Adolescents repeatedly stated that racism is a part of the world we live in and is unchangeable. One participant’s statement that racism is “another day in the life” was met with a chorus of nodding and replies of “another day.”

#### Subtheme 2: Dependent on the Target of Racism

Many adolescents identified circumstances in which they experienced more feelings of helplessness based on who the target of racism was. Specifically, several adolescents reported difficulty coping with racism directed at their friends, family, or themselves, whether in-person or online. There was some disagreement within focus groups, with other participants reporting feeling more helpless regarding racist events involving those they did not know. Participants endorsed feeling a greater sense of control when racism was directed at them but felt relatively powerless when racism was directed at others. During 1 focus group, when asked about racist events, participants brought up the Jason Van Dyke trial, which had reached a verdict a few months before the group met. Jason Van Dyke was a Chicago police officer sentenced to 81 months in prison after fatally shooting Black adolescent Laquan McDonald.^12^ Participants recalled the consequences of the court ruling and how they felt. Of note, African American/Black and Hispanic/Latinx participants still felt a sense of loss of control and helplessness even with the court ruling against Van Dyke.

#### Subtheme 3: Futility in Responding

The perceived ability or inability to change a situation contributed to adolescents’ feelings of helplessness. These feelings, in turn, affected how participants responded to racist material: helplessness was cited as the main reason they chose not to respond at all. Adolescents across the focus groups had different explanations for their feelings of helplessness, including the events being difficult to process, the frequency of these events, and, finally, the uncertainty and anger about how to enact change. While helplessness was 1 response to vicarious racism, participants exhibited other responses, like activism.

### Coping Response: Activism

Participants were asked about positive coping mechanisms to deal with racist events online, and they identified the people, including family and friends, with whom they confide, grieve, and find support. Adolescents reported that activism helped them cope with the world around them. Activism is defined here as taking action collectively or individually to bring about meaningful change to address issues of social justice,^13^ which in this case would benefit people marginalized on the basis of their race or ethnicity. Although participants were not explicitly asked about activism, this theme formed a common thread across focus groups, as participants described their responses to media-based racism, with 3 subthemes (Table 3). They described both activism at an individual level (ie, online) and as part of a group.

**Table 3.** Activism Theme and Examples of the Subthemes from Adolescent and Young Adult Participants

Theme	Subtheme	Examples of statements introduced by participants
Activism	Activism as altruism	“I feel like if we have the right coping mechanisms and like the right ways to deal with it then we can use those negative emotions for like good, you know because then we can be more passionate and like change our actions to spread … loving awareness for like these kinds of things.”
		“Like I might repost something that I feel passionate about but like I also try to spread things like positivity and like happiness and like I don’t know because in a way it makes me feel better. But I also want other people to know that like it’s not all bad. And I don’t want everyone to be discouraged because then nothing will get fixed.”
	Online activism	“There was a time that somebody I know on his Instagram story, he posted like he was on a plane and I guess there was an Indian-Pakistani man and he took a picture of him and was like ‘I’m scared.’ I let him know that that was very ignorant, and then I posted it on my story, and made a comment about it being ignorant as well.”
		“Sometimes you just can’t accept it, and arguing with somebody is not wrong. It’s just how you argue [that matters].”
	Collective activism	“It’s funny because a lot of people think that teenagers are not socially aware, but I think the friends that I keep around me, once we start talking about something it goes on and on and on and on. Being in programs that allow you to express yourself and actually talk to other people about how you feel on certain issues actually I feel like help everyone get a greater view and perspective on the different minds that we do have and how to accept how other people feel.”
		“Yeah, and then sometimes my response is it’s something I can do something about, like right now. ‘Oh, hey this thing is happening with police. No Cop Academy [a Chicago campaign to urge the city to invest money into marginalized communities instead of a new police training academy] meeting is next week.’ I was like, ‘I’m going.’ Or I’m sending support to one of my orgs that is actively going against whatever just happened.”

#### Subtheme 1: Activism as Altruism

Participants spoke about altruism and wishing to engage in action or activism that helped others, which emerged as a subtheme. Adolescents wished to bring about change to improve their futures and verbalized the importance of reaching out to lift up their peers. Adolescents stated that while they could not always make vast changes to systems, helping peers through activism helped them as well.

#### Subtheme 2: Online Activism

Many participants’ examples of activism involved action online, such as reposting on social media, given the pervasive role of the internet in their lives and interactions as well as the study’s explicit emphasis on racism online. Participants discussed factors that informed their decision to comment on or share stories on social media. Many adolescents reported choosing to share stories with their friends for private discussion, either through direct message (DM) functions within social media platforms or personal text messages. Fewer instances were described in which they sent DMs to the original person who posted a story (the poster) if they wished to discuss content they found racist. While a few participants reported that they had made a point to publicly address an original poster, this was rare because adolescents preferred not to engage in so-called comment wars and further perpetuate their feelings of helplessness or futility. When they did publicly address the poster, usually they would do so when the event either directly involved themselves or their friends. Participants discussed a certain tipping point, where such content needed to be addressed, and they placed emphasis on how to address these individuals.

#### Subtheme 3: Collective Activism

Although discussed less often, in-person activism was another tool adolescents reported using to exercise their demands for change, including group participation through school or community programs. Participants specifically discussed youth groups that they belonged to, where they could discuss topics like improving school environments for students of color (eg, Asian and Black students). Adolescents discussed issues they hoped to bring to the attention of school administration to achieve a better school environment and effectively promote change. Also, participants discussed affecting change within their own communities in Chicago. They considered the utility of getting involved in what was going on around them, either through doing the work themselves or by helping organizations that engaged in work they supported. Overall, adolescents vocalized their desire to channel their negative emotions into positive action, but they also recognized the importance of grieving and feeling pain caused by racist events. Participants reflected on how these events can actually bring them closer together as a community to continue to persevere.

Finally, participants did not identify a universal reason for engaging in activism. A participant’s tipping point into activism was guided by the context of the situation as well as the accessibility and ease of the action and weighed against the benefits and drawbacks of action. Furthermore, while participants discussed the negative emotions and coping strategies used, they had more difficulty drawing firm connections to other aspects of health or health behaviors (eg, exercise, diet, taking medications).

## Discussion

In this adolescent-centered qualitative study, we found that participants reported feeling helplessness after exposure to media-based vicarious racism and used activism as a main source of coping. Our findings explore how systemic racism contributes to helplessness and how teens engage with activism to channel their negative emotions into action. Although prevention and intervention strategies that limit racism exposure and thwart subsequent harms can be difficult to identify, adolescents reported using unique coping strategies to combat structural racism and create change within and outside of their communities.

Helplessness was described as an initial response to exposure to media-based vicarious racism and is aligned with prior work, which describes adolescent depressive symptoms and negative emotion cycling in response to exposure to racist material.^7,14^ Helplessness contributed to their feelings of racism as an immutable part of the world. However, their feelings of helplessness may be dependent on the closeness of the initial target of racism. Participants identified the greatest feelings of helplessness when they or those within their social network experienced racism. This may signify that exposure to vicarious racism is associated with a greater emotional response for those in our participants’ social network. This may be due to the social cohesion and emotional connection of those in an adolescent’s social network. However, the social network may not be the only important characteristic that affects the emotional impact vicarious racism has on adolescents. Extant literature has postulated that if an individual identifies with the intended target of racism, they may be more affected by the event than if they do not identify with the target.^6^ Specifically, individuals with the same race/ethnicity as the victims or survivors of racist acts may have more negative mental health symptoms after exposure to large-scale events of racism. For example, a recent study found that exposure to police killings of unarmed Black US residents was associated with more days of reported poor mental health among US Black participants.^5^ Additionally, helplessness also contributed to a participants’ decision to act or not. Furthermore, helplessness may affect adolescent physical health, mental health, and well-being.^15,16,17,18,19^ However, some adolescents can catalyze their emotions and transform them into action.

Although online environments provided a primary avenue for vicarious racism exposure, it also granted easy access to online activism, given the frequency with which teens were exposed to racist content through social media. Activism may be most comparable with problem solving, which is a well-studied coping mechanism.^20,21,22,23^ Activism allows adolescents to engage in solution-oriented work and grants them a sense of control. Activism, the act of doing something, may lessen the feelings of helplessness (“there’s nothing I can do”). Participants described why they engaged in activism (eg, altruism, social support) and which type of activities they choose (eg, online or in-person). Participants stated that activism can create better environments for themselves and their peers, and prior research demonstrates improved outcomes in health and well-being.^24^ Participants’ initial responses to racist content included reposting material or commenting on others’ posts, but they did not discuss participation in other online activities or platforms (eg, fundraising, petition signing, or online discussions), which may embody a more robust set of activism activities.^25^

Participants expressed a mature understanding of the sociopolitical climate surrounding them. Participants sought out opportunities for in-person activism, giving them a sense of collective efficacy, through their schools and community organizations. Specifically, collective efficacy is the willingness of an individual to intervene on behalf of the good of their respective community.^26^ The willingness to intervene is paired with the belief that these actions can create change within their communities. Furthermore, participants felt a desire to create change within their local community, as local issues were more closely tied to their social networks of families, friends, and peers. These social ties and collective activities may be helpful in combating helplessness. This study aligns with previous studies that demonstrate social cohesion or connectedness can buffer the negative effect racism has on health generally, and the benefits of activism may be because adolescents can connect to a larger social network to draw support and participate in collective action.^2,3^ Additionally, studies suggest that activism may moderate negative mental health symptoms for African American and Latinx adolescents.^13,27^

Adolescents are not immune to the harms of racism and can experience racism online, whether they are the intended target or not. Adolescents have ready access to internet and, thus, exposure to media-based racism online. Exposure to vicarious racism online, regardless of the type of content or intended target, elicited helplessness and also prompted activism in many of our participants. Investigating adolescents’ exposure and response to racism online proves critical because of the negative changes in emotional state and potential adverse physical and mental health outcomes across the life course. Our participants did not directly associate exposure to media-based racism with changes in their physical health or health behaviors but discussed their emotional health. This study specifically focused on the adolescent experience to offer invaluable insights on navigating contemporary racism. The findings suggest adolescents exhibit helplessness and activism, seemingly opposite responses to vicarious racism. However, racism is a complex issue that will produce complex emotional and coping responses. Activism may provide adolescents the opportunity to process their feelings in a healthy manner. Future studies should build on these findings and, perhaps, develop and test interventions aimed at reducing feelings of helplessness and empowering adolescents affected by media-based racism to participate in antiracist activism.

### Limitations

Our study should be interpreted within the context of its limitations. First, our sample was limited by the lack of sex and gender diversity. Of note, we did not report participant gender identity to maintain anonymity for gender minority youth; however, our focus groups had relatively fewer male participants. To further the field, there is a need for research that intentionally includes a gender-diverse sample to make our results more generalizable. Future research should examine the type of media-based vicarious racism content and responses to vicarious racism analyzed by demographic identities (eg, gender, race, and socioeconomic status) and the intersection of those identities (eg, intersectionality).^28^

Additionally, our participants may have been more aware of racism and more prone to action due to our recruitment strategy. Many adolescents in our sample were involved in youth groups in school that discussed racism and bias, which may not represent the greater Chicago adolescent population. However, we expect our population would have similar exposures to media-based vicarious racism, and given their involvement in youth groups, they may better describe some of the key aspects of activism that adolescents experience.

As with any focus group–based research, social desirability bias should be considered.^6^ Specifically, participants may have been less likely to state dissenting opinions. Additionally, we were unable to analyze specific media-based racist content participants consumed (eg, news articles, stories, videos, and comments) or present additional coping mechanisms, such as social support, because this was out of the study’s scope. Future research should consider the use of community-based participatory research designs, as that may inform the study design and subsequent results, given that adolescents would be able to better guide the scholarship.

## Conclusions

In this qualitative study, adolescents reported experiencing helplessness as a primary emotion after exposure to media-based vicarious racism, and they endorsed activism as means to exercise their own power in the face of persistent structural racism. The summer of 2020 was characterized by a wave of racialized violence against Black individuals in the US that captured national attention. The deaths of George Floyd, Ahmaud Arbery, and Breonna Taylor were featured in the news, in part due to sustained and widespread protests and public outrage. National news coverage revealed not only the problematic and systemic role of racism in the lives of individuals from racial/ethnic minority groups but also illustrated that activism is a powerful response to that racism. Adolescents are astutely aware of racism and have a nuanced view of how it affects them and others. Long-lasting societal transformation, including the elimination of health disparities, will require antiracist interventions. Multilayered approaches, including peer and family support, may be needed to buffer adolescents’ exposure to racism online, specifically strategies that decrease helplessness and encourage activism safely. While the onus of addressing racism should not rest on adolescents, adolescents who chose to participate in activism may be able to reduce their own negative emotions, support the emotional and mental health needs of others, and create the public will needed for policy change.

## Notes

Supplement. **eAppendix.** First Author Perspective eReferences.
