# Supplementary material for: Adolescents’ Experiences, Emotions, and Coping Strategies Associated With Exposure to Media-Based Vicarious Racism
Source: JAMA Netw Open. 2021 Jun 15;4(6):e2113522. doi: 10.1001/jamanetworkopen.2021.13522 (PMC8207240; doi:10.1001/jamanetworkopen.2021.13522)
Supplement: Supplement. — eAppendix. First Author Perspective eReferences. [file jamanetwopen-e2113522-s001.pdf]

Supplemental Online Content

Heard-Garris N, Ekwueme PO, Gilpin S, et al. Adolescents' experiences, emotions, and coping strategies associated with exposure to media-based vicarious racism. *JAMA Netw Open.* 2021;4(6):e2113522. doi:10.1001/jamanetworkopen.2021.13522

eAppendix. First Author Perspective

eReferences.

This supplemental material has been provided by the authors to give readers additional information about their work.

© 2021 Heard-Garris N et al. *JAMA Network Open.*

eAppendix 1. First Author Perspective-

Dr. Nia Heard-Garris is the first author of the manuscript entitled, "'Sometimes you just can't accept it': Exploration of Exposure to Media-based Vicarious Racism, Emotions and Coping Strategies Among Adolescents."

I identify as a feminine-presenting, Black American physician-scientist. As a pediatrician, I focus on the influence of social stressors on child and adolescent health. This work includes structural racism's impact on pediatric health. I fully acknowledge that both my personal and professional experiences have deeply contributed to my interest on this work. When I engage in scholarship that revolves around racism, I do so with intentional reflection. This practice is important in all forms of research but particularly with respect to qualitative research. In the article, Reflectivity in Research Practice: An Overview of different perspectives, Mortari notes that researchers should make the "politics of research transparent" and thus should not only report the research, but also question how the findings came to be.[1] This is because researchers do not "merely reflect the phenomenon, but he or she shapes it;" therefore, "there is no possibility of an objective science."[1] I have always believed that science could never be truly "objective" and have tried to understand my own biases when engaging in research. Particularly, many have said that people of color, here, a Black person in the United States cannot truly study racism objectively, given the Black experience in the US. However, I think people of color are able to ask and seek out the questions and answers that scholars who are not of color would never think to ask because of their own lived experiences. This then, contributes to a more holistic view of these sociological phenomenon. With respect to this study, I can absolutely identify with feelings of helplessness in response to racism in all forms. Specifically, I often cite my commitment to this work stemming from an upsetting experience in residency, where I was pregnant with my son and the murder of Trayvon Martin, a Black teenager had recently occurred. His death impacted me and began my quest to study and better understand experiences of vicarious racism. Because of my personal experiences and the experiences of my team, it could be possible that the research team looked for instances of helplessness and focused in on these themes and overlooked others. However, this is one important reason to have diverse research teams. This particular study team had a diverse racial and ethnic makeup, including a Black American (an American Descendent of slaves), a first generation Pakistani American, a first generation Jamaican American, a second generation Nigerian American, and white Americans. All our experiences with racism and discrimination were varied. Some of us had experiences encountering discrimination because of our religious faith, nativity, skin color, or no experiences of note. Further, while we entered in this work hoping to understand more about racism, we tried to remain open as to what we may find. Activism, in particular was a surprise, as it was an emergent theme. One of the reasons we chose to highlight activism was because it was a hopeful act that adolescents did in response to racism, so that we were not only presenting helplessness or apathy. Finally, as is often the case, qualitative work leads us to want to ask more questions and desire to learn more about what we find and because of this initial study, we have a solid understanding of where to go next.

© 2021 Heard-Garris N et al. *JAMA Network Open.*

eReferences

1. Mortari L. Reflectivity in Research Practice: An Overview of Different Perspectives. *Int J Qual Methods*. 2015;14(5):1609406915618045. doi:10.1177/1609406915618045

© 2021 Heard-Garris N et al. *JAMA Network Open.*
